# Accompanying your children: Living without parents at different stages of pre-adulthood and individual physical and mental health in adulthood

**DOI:** 10.3389/fpubh.2023.992539

**Published:** 2023-03-13

**Authors:** Yao Jiang, Hanling Xiao, Fan Yang

**Affiliations:** ^1^Department of Demography, Zhou Enlai School of Government, Nankai University, Tianjin, China; ^2^Department of Labor and Social Security, School of Public Administration, Sichuan University, Chengdu, Sichuan, China

**Keywords:** left-behind children, physical health, mental health, family structure, parental absence

## Abstract

**Objectives:**

This study examined how living without parents at different stages of childhood and adolescence affects physical and mental health in adulthood.

**Methods:**

The data came from 3,464 survey respondents aged 18–36 in the 2018 China Labor-Force Dynamics Survey. Physical health was self-rated. Mental health was measured by the Center for Epidemiological Studies Depression scale. The ordered probit and ordinary least-squares regression analyses were employed to determine the associations between growing up without parents at different stages in pre-adulthood and individual physical and mental health in adulthood.

**Results:**

Individuals who did not live with their parents during their minor years were more likely to report worse physical and mental health in adulthood, compared to those who lived with their parents. This difference was heterogeneous among different age stages and genders.

**Conclusions:**

Absence of parents in the household has long-term impacts on the physical and mental health of children in adulthood, especially for females. The government should make feasible institutional arrangements to avoid the separation of minor children from their parents.

## Introduction

Numerous factors during childhood and adolescence have significant impact on an individual's health in adulthood (1, 2). Current studies mainly focus on how the health of caregivers is impacted by caring for children and adolescents (3), or on the current health of children and adolescents (4–12). Research has not yet highlighted how parental absence at various stages of childhood and adolescence can affect physical and mental health in adulthood.

The situation of minors living without parents is generally due to parental divorce, death, or working away from the home (13–16). Based on the attachment theory (17), parental absence may harm children's physical and social psychological development and lead to negative outcomes eventually (18). As an emotional bond of one person with another person, the behavior of attachment is a necessary psychological need that human beings are born with (19). The early emotional bonds formed by children with their caregivers (mainly parents) have significant impacts on children's cognitive and socioemotional development throughout life (20–22). The attachment system serves two primary functions by providing instrumental and emotional support (23). One is to protect individuals from potential threats or injuries, and the other is to regulate individual negative emotions following threatening or harmful events (23). Children who maintained proximity to an attachment figure were more likely to receive care, comfort and protection (17). If the attachment is lost or weakened such as parental absence, it may be detrimental to the physical and mental development of children and ultimately affect physical and mental health of children for a long time (17–23).

For the physical health, a study by Schwartz and McLanahan suggested that absence of the father during children's growing years can result in poor physical health outcomes to the children (13). Whereas, the father, as the main provider of income for a family, determines the quality of child care and health care that children receive. The absence of the father may lead to poor care for the child, and may result in insufficient food and nutrition for the child, which may have negative impacts on the child's physical health (13).

For the mental health, the economic hardship of a single-parent household may cause depression and psychological distress in children (13). Compared to non-bereaved children, children who lost a parent to death showed more serious mental illness (14). In the first 2 years following the death of a parent, children experienced increased risk of psychiatric disturbance (15). In a survey of children with multinational family backgrounds, it was found that compared with children living with both parents, children in households where the father was absent due to migrant work in Indonesia, Vietnam, and Thailand had greater odds of experiencing emotional disorders (16).

The absence of parents affects not only the physical and mental health of minors as they are growing up, but also their physical and mental health in adulthood (24–28). From adolescence through early adulthood, individuals from non-intact families are more likely to engage in adverse health-related behaviors including smoking, alcohol consumption, poor nutrition habits, and low physical activity, compared with those who grow up in intact families (24). They also have worse self-reported health and more subjective health complaints. Temporary parental separation very soon after birth can have unfavorable effects on later psychological development, including vulnerability to addiction (25) and a certain degree of depression risk (26). For instance, according to a 28-year cohort research which was consisted of 3,020 subjects in Finland, the 4% of adult respondents who experienced temporary separation at birth had been treated in hospital due to a depressive episode, and the incidence was higher than that of respondents who did not experience temporary separation at birth (26). Parental divorce can negatively affect the mental health of young adults (27). However, experience of parental divorce in childhood may not be an indicator of adult psychiatric or somatic health issues (28). Overall, although prior studies have examined the effects of absence of parents on current health of children or their health in adulthood, a consensus has not been reached.

As the main reason for separation of children from their parents in China is that the parents leave rural areas to go to work in cities, the left-behind children are the main component of the kids who did not spend childhood or adolescent with their parents (29). As of 2020, the number of children left behind in rural China totaled 6.436 million (29). While their parents work in cities, these so-called left-behind children may live with their grandparents, older brothers and sisters, other relatives, or alone. According to China Ministry of Civil Affairs, 96% of these left-behind children aged under 16 years old are lived with their grandparents (30).

Children who are left behind have lower levels of physical and mental health than their peers (31). The adverse effects of lack of parental care and attention tend to accumulate over time (32–34). Left-behind children are shorter than their peers due to insufficient intake of energy, protein, calcium, and other nutrients (32). They may be at higher risk for stunted growth, unhealthy food preferences, lower physical activity, smoking, alcohol consumption, injuries, and incomplete vaccination (32). In addition, they are more prone to negative emotions, social anxiety, and low self-esteem (33, 34). However, on the positive side, children's health and experiences may benefit from the greater income earned by parents working abroad or in cities (35, 36).

On the whole, the parental absence harms more than it benefits the physical and mental health of minor children. Although some studies have shown that the effect of parental absence on children's health is lasting, its effect at different ages on children's physical and mental health in adulthood has not been widely explored. According to developmental psychology, minors have different developmental needs at different age stages (37, 38). Therefore, children's needs on parental accompany in different age stages before adulthood may be heterogeneous (39–42). For example, at the age of 0–6, children may mainly need material care and emotional companionship from their parents (39); for children aged 7–12, the development of living habits needs to be carried out under the supervision of their parents (40, 41); when children are 13–15 years old, they generally enter a rebellious period, and parents need to help them deal with emotional problems in this stage (42). Generally, compared with children whose parents are not absent, children whose parents are absent are less likely to be observed and satisfied with their development needs at different age stages before adulthood. This is more likely to have a negative and lasting impact on children's physical and mental health. Furthermore, as children growing older, they tend to be independent of their parents and the early attachment between children and parents may be gradually weakened (43). Thus, the effect of parental accompany before adulthood on children's health outcomes in adulthood may present a decreasing trend. However, few studies have made attempts to empirically test the effect of parental absence at different age stages on children's health in adulthood.

In this paper, we divided period before individual adulthood into four stages based on Chinese situation-−0–6, 7–12, 13–15, and 16–18 years old. In general, 0–6 years old is a stage of preschool age in China, 7–12 years old is primary education stage, 13–15 years old is a stage of secondary school, and 16–18 years old is high school stage. This age division based on Chinese educational regime appropriately covers all stages of an individual before adulthood (44). It also reflects the physical and mental development characteristics of individuals at different age stages before adulthood to a certain extent.

Moreover, gender is a vital social lens to promote the more careful and targeted child care (45). Compared with boys, girls are more vulnerable to the inequality of being cared for before adulthood (46). As mentioned above, the vast majority of left-behind children (96%) live with their grandparents in China. These older grandparents are more influenced by the traditional patriarchal ideology than younger parents to a large extent, and they may take less care of female grandchildren than male grandchildren (47, 48). Therefore, the absence of parents may have a greater negative impact on the physical and mental health of girls than boys. However, few studies have made attempts to test the heterogeneous influences of living without both parents during childhood and adolescence on individuals' physical and mental health in adulthood from the perspective of gender difference.

**Figure 1:**
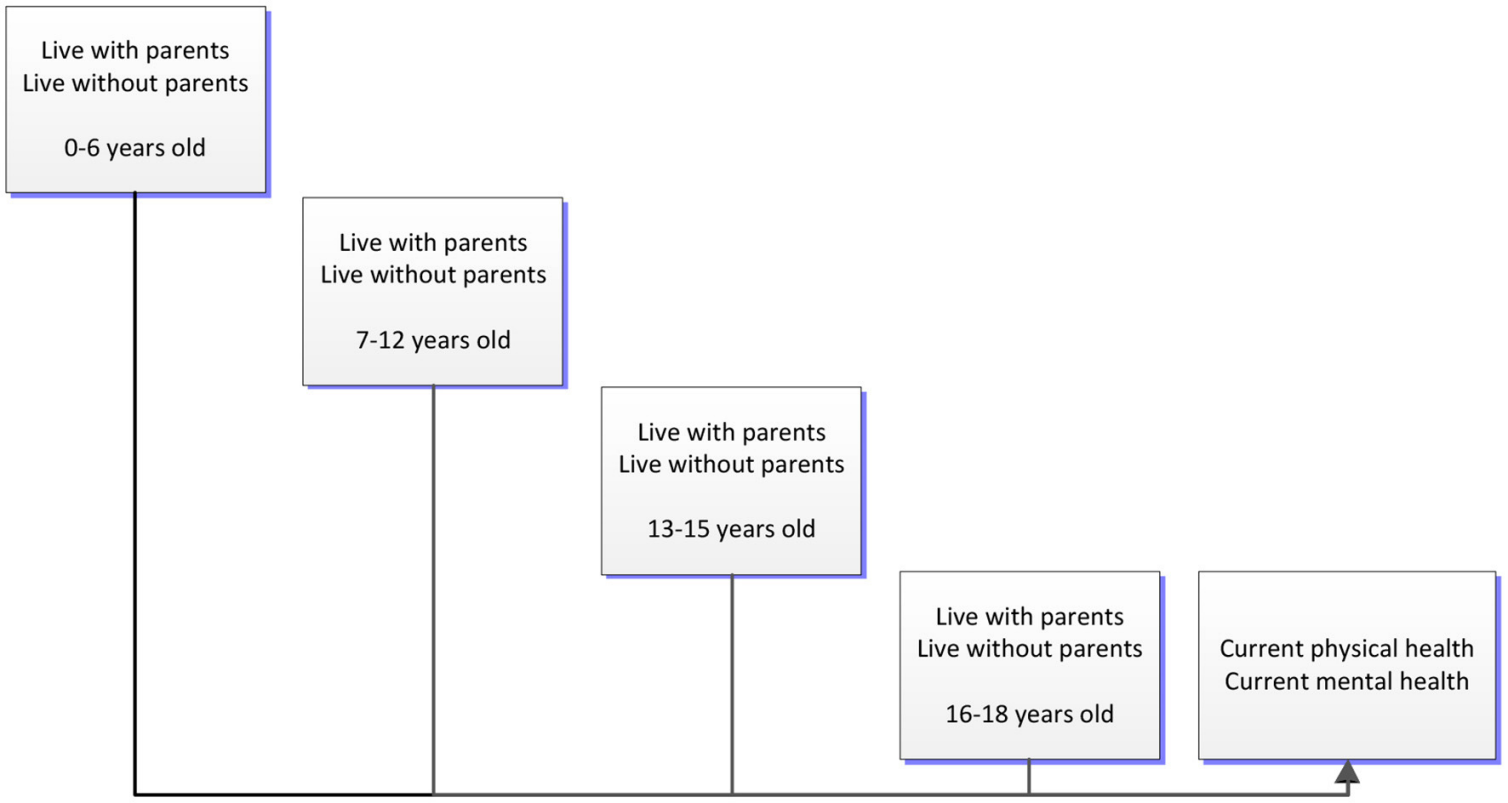
Study framework.

Hence, in this study, we used a nationally representative survey of 3,464 Chinese respondents with an average age of 28 years, and expanded the body of knowledge of this subject by focusing on the long-term effects of living without parents at different age stages before adulthood. We observed how living without parents during the age ranges 0–6, 7–12, 13–15, and 16–18 affected the physical and mental health of individuals in adulthood (Figure 1). In addition, we conducted a heterogeneity analysis based on gender. Our study should be of interest to researchers and public policy makers concerned with the welfare of children and adolescents.

## Methods

### Data source

Our study used data from the 2018 China Labor-Force Dynamics Survey, a comprehensive study conducted by the Center for Social Science Survey at Sun Yat-sen University to collect information on Chinese education, work, migration, health, economic activities, and other interdisciplinary aspects (49). The major objective of the survey was to provide basic public data for social science research in China. It was designed using a multistage cluster and stratified probability-proportional-to-size sampling strategy, and computer-assisted interviews were conducted in respondents' homes or by telephone. It collected data from 29 provincial administrative units across the country, so it is nationally representative. After cleaning the data by excluding the missing values, outliers, and other abnormal values, we obtained useful samples from 3,464 respondents. Because the data were collected by professionals at the university, its validity is assured.

### Participants

Among the analysis samples of this paper, males represented 45.4% of the total participants. The age of the participants ranged from 18 to 36 years, with a mean value of 28.225 (SD = 5.206). The average years of school were 11.523 (SD = 3.913), between junior high school and senior high school. The logarithm of total annual income of the participants in 2017 had a mean value of 10.533 (SD = 0.878), with a minimum value of 5.704 and a maximum value of 14.914. A total of 70.2% of the participants were married, 21.8% had the habit of smoking, 16.9% drank alcohol, and 36.4% exercised regularly.

### Measures

#### Explained variables

Physical and mental health were the two explained variables in our analysis. Physical health status was self-rated (50–53). Respondents were asked “How would you evaluate your current health status?” and could rate their responses from 1 to 5 on a five-point Likert scale that included “very unhealthy,” “somewhat unhealthy,” “normal,” “somewhat healthy,” and “very healthy.”

The Center for Epidemiological Studies Depression (CES-D) scale developed by Radloff was employed to assess the mental health of respondents (54, 55). The CES-D is one of the most widely used scales for measuring depression and mental health (56, 57). It has been verified as valid for the assessment of depression and mental health status in a Chinese context (58–62). The CES-D scale is scored from 20 to 80, and a higher score indicates a higher level of depression and worse mental health (54). The Cronbach's alpha on CES-D scale is 0.949 in this study.

#### Explanatory variables

The explanatory variables represented who the individual lived with at specific age ranges while growing up. Respondents were asked to recall, “Who did you live with when you were 0–6 / 7–12 / 13–15 / 16–18 years old?” If a child was living with both parents during a specific age range, the response was assigned a value of 0; other responses received a value of 1.

#### Control variables

To adjust for potential confounding effects on associations between living without parents at different stages and individual physical and mental health in adulthood (63–66), we used several control factors in our regression model analyses. The variables used were gender (male = 1, female = 0); age in years; number of years of schooling; marital status (married = 1, unmarried = 0); logarithm of the total annual income of the respondent in 2017; smoking (habitual smoking = 1, otherwise = 0); drinking (habitual drinking = 1, otherwise = 0); and exercise (habitual exercise = 1, otherwise = 0). In addition, we controlled for regional effect based on the provinces where respondents were located.

### Analysis strategy

Descriptive statistics were computed to estimate the proportion of respondents living with and without parents at each age range, the current physical and mental health status, and the demographic characteristics of the respondents. In models for different age ranges of living without parents, multivariable analyses adjusted for the confounding effects of general factors affecting individual health in adulthood (67). We used ordered probit regression models to analyze the effects of living without both parents at different stages on the physical health of individuals in adulthood. The main equations for this specification can be written as follows (68):


(1) $$physical\;health_{i}^{*}=\beta^{\prime }x_{i}+\varepsilon_{i}$$

where *i* denotes an individual observation, $physical\;health_{i}^{{\;}^{*}}$ represents the unobserved dependent variable, viz., individual's physical health, *x*_*i*_ represents a vector of explanatory variable, *β*′ represents a set of parameters, and *ε*_*i*_ is a random error term which is followed normal distribution. In general, $physical\;health_{i}^{{\;}^{*}}$ cannot be observed directly, but the categorical variable *physical health*_*i*_ can be observed. The relationship between $physical\;health_{i}^{{\;}^{*}}$ and *physical health*_*i*_ can be represented as follows:


(2) $$\begin{array}{l} physical\;health_{i}=1\;if0\leq physical\;healthi_{i}^{*}\leq \;\mu_{1}\\ physical\;health_{i}=2\;if\mu_{1}\leq physical\;healthi_{i}^{*}\leq \mu_{2}\\ \;\vdots \\ physical\;health_{i}=M\;if\mu_{M-1}\leq physical\;healthi_{i}^{*} \end{array}$$

where μ are the cut points, which are the unknown parameters to be estimated along with *β*′, and *M* are the possible outcomes for *physical health*_*i*_. In this study, *M* ranges from “1” to “5.”

To estimate the effects on individual mental health in adulthood, we used ordinary least-squares regression models. The specific ordinary least-squares model is as follows:


(3) $$\begin{array}{l} mental\;health_{i}=\alpha_{0}+\alpha_{1}x_{i}+\alpha_{2}X+\varepsilon_{i} \end{array}$$

where *mental health*_*i*_ represents the explained variable of mental health which is measured by the CES-D scale, and *x*_*i*_ means the explanatory variable. α_0_ denotes the intercept item, α_1_ and α_2_ are coefficients for explanatory variable and control variables, respectively. *X* means a set of control variables, and *ε*_*i*_ is the random error item.

Furthermore, there may be some observable systematic differences among individuals, and it is the respondents' family self-selection to live with parents or not. If we compared the two groups of respondents directly, the estimation results may be biased due to the self-selection of samples. Thus, to confirm the influences of living without parents at various stages of childhood on individual physical and mental health in adulthood, the propensity score matching (PSM) method was used to build a counterfactual framework. The main equations of PSM model can be written as follows (69):


(4) $$\begin{array}{l} ATT_{p}=E\left[\left(physical\;health_{1i}-physical\;health_{0i}\right)\text{|}AS_{i}=1\right]\\ \;=E\{E\left[\left(physical\;health_{1i}-physical\;health_{0i}\right)\text{|}AS_{i}=1\right],\\ \;p(Z_{i})\}\\ \;=E\{E\left[physical\;health_{1i}\text{|}AS_{i}=1\right],\;p(Z_{i})\\ \;-E\left[physical\;health_{0i}\text{|}AS_{i}=0,p(Z_{i})\right]\text{|}AS_{i}=1\} \end{array}$$

(5) $$\begin{array}{l} ATT_{m}=E\left[\left(mental\;health_{1i}-mental\;health_{0i}\right)\text{|}AS_{i}=1\right]\\ \;=E\{E\left[\left(mental\;health_{1i}-mental\;health_{0i}\right)\text{|}AS_{i}=1\right],\\ \;p(Z_{i})\}\\ \;=E\{E\left[mental\;health_{1i}\text{|}AS_{i}=1\right],\;p(Z_{i})\\ \;-E\left[mental\;health_{0i}\text{|}AS_{i}=0,p(Z_{i})\right]\text{|}AS_{i}=1\} \end{array}$$

Where *ATT*_*p*_ and *ATT*_*m*_ are the average effect of treatment on the treated. *physical health*_*i*_ and *mental health*_*i*_ are the explained variables, *AS*_*i*_ denotes a binary treatment variable, specifically, taking a value of “1” for respondents who lived without both parents at 0–6 / 7–12 / 13–15 / 16–18 years old; otherwise, *AS*_*i*_ = 0. *p*(*Z*_*i*_) represents the propensity scores estimated by PSM estimation, and *Z*_*i*_ represents a set of covariates.

## Results

### Descriptive statistics

Table 1 reports the definitions of the variables employed in this study and the results of the descriptive analysis (*n* = 3,464). Of the explained variables, the average value for respondents' physical health was 3.995 (SD = 0.820) on the five-point Likert scale ranging from 1 to 5, which means the physical health of the respondents was generally between “normal” and “somewhat healthy.” The average value for mental health of respondents was 26.858 (SD = 8.217) on the CES-D scale ranging from 20 to 80.

**Table 1:** Descriptive statistics (*n* = 3,464), China, 2018.

**Variable**	**Definition**	**Mean**	**SD**	**Min**	**Max**
**Explained variable**					
Physical health	1 = very unhealthy; 2 = somewhat unhealthy; 3 = normal; 4 = somewhat health; 5 = very healthy	3.995	0.820	1	5
Mental health	Total score of the CES-D ranges from “20” to “80.” The higher CES-D score, the deeper depression, and the worse mental health	26.858	8.217	20	80
**Explanatory variable**					
Living without parents (0–6 years)	0 = living with parents; 1 = living without parents	0.096	0.295	0	1
Living without parents (7–12 years)	0 = living with parents; 1 = living without parents	0.104	0.305	0	1
Living without parents (13–15 years)	0 = living with parents; 1 = living without parents	0.140	0.347	0	1
Living without parents (16–18 years)	0 = living with parents; 1 = living without parents	0.219	0.413	0	1
**Control variable**					
Gender	1 = male; 0 = female	0.454	0.498	0	1
Age	Years old	28.225	5.206	18	36
Education	Years of schooling education of respondent	11.523	3.913	0	23
Marital status	1 = married; 0 = unmarried	0.702	0.457	0	1
Logarithm of income	Logarithm of total annual income of respondent in 2017	10.533	0.878	5.704	14.914
Smoking	1 = have habit of smoking; 0 = else	0.218	0.413	0	1
Drinking	1 = have habit of drinking; 0 = else	0.169	0.375	0	1
Exercise	1 = have habit of exercise; 0 = else	0.364	0.481	0	1

The explanatory variables, either living with parents (represented by a value of 0) or without parents (represented by a value of 1) before adulthood, were stratified into four different age stages. Of the total respondents, 9.6% lived without their parents during the ages of 0–6, 10.4% during the ages of 7–12, 14% during the ages of 13–15, and 21.9% during the ages of 16–18.

### Benchmark regression

Table 2 reports the ordered probit model and the ordinary least-squares model results. It can be observed from Table 2 that compared to individuals who lived with their parents, individuals who did not live with their parents during the ages of 0–6 had significantly worse physical health in adulthood (coefficient = −0.223, *p* < 0.01). A similar situation also occurred in the age ranges 7–12 (coefficient = −0.169, *p* < 0.01), and 13–15 (coefficient = −0.099, *p* < 0.1). However, there were no significant difference in the effects of living with and without parents at the ages of 16–18 on the physical health of individuals in adulthood.

**Table 2:** Influences of living without parents at different stages before adulthood on individual physical and mental health in adulthood, China, 2018.

**Variables**	**Physical health (ordered probit)**				**Mental health (OLS)**			
	**(1)**	**(2)**	**(3)**	**(4)**	**(5)**	**(6)**	**(7)**	**(8)**
Living without parents (0–6 years)	−0.223^***^				2.632^***^			
	(0.064)				(0.471)			
Living without parents (7–12 years)		−0.169^***^				2.178^***^		
		(0.061)				(0.454)		
Living without parents (13–15 years)			−0.099^*^				1.355^***^	
			(0.054)				(0.402)	
Living without parents (16–18 years)				−0.022				0.696^**^
				(0.046)				(0.340)
Gender	0.026	0.024	0.025	0.024	−0.817^**^	−0.791^**^	−0.803^**^	−0.784^**^
	(0.047)	(0.047)	(0.047)	(0.047)	(0.345)	(0.345)	(0.346)	(0.346)
Age	−0.017^***^	−0.016^***^	−0.016^***^	−0.015^***^	0.048	0.042	0.040	0.034
	(0.004)	(0.004)	(0.004)	(0.004)	(0.032)	(0.032)	(0.032)	(0.032)
Education	0.032^***^	0.031^***^	0.031^***^	0.031^***^	−0.180^***^	−0.175^***^	−0.172^***^	−0.173^***^
	(0.006)	(0.006)	(0.006)	(0.006)	(0.042)	(0.042)	(0.042)	(0.042)
Marital status	0.088^*^	0.088^*^	0.088^*^	0.087^*^	−1.147^***^	−1.150^***^	−1.145^***^	−1.141^***^
	(0.050)	(0.050)	(0.050)	(0.050)	(0.369)	(0.369)	(0.370)	(0.370)
Logarithm of income	0.046^*^	0.045^*^	0.045^*^	0.045^*^	−0.216	−0.207	−0.212	−0.203
	(0.024)	(0.024)	(0.024)	(0.024)	(0.177)	(0.177)	(0.177)	(0.177)
Smoking	0.072	0.073	0.073	0.071	−0.512	−0.521	−0.527	−0.517
	(0.057)	(0.057)	(0.057)	(0.057)	(0.421)	(0.421)	(0.422)	(0.422)
Drinking	−0.009	−0.005	−0.005	−0.005	1.222^***^	1.181^***^	1.172^***^	1.161^***^
	(0.057)	(0.057)	(0.057)	(0.057)	(0.420)	(0.420)	(0.421)	(0.422)
Exercise	0.084^**^	0.085^**^	0.086^**^	0.086^**^	−0.032	−0.046	−0.062	−0.069
	(0.041)	(0.041)	(0.041)	(0.041)	(0.301)	(0.302)	(0.302)	(0.303)
Region	Yes	Yes	Yes	Yes	Yes	Yes	Yes	Yes
*n*	3,464							
Pseudo R^2^	0.045	0.044	0.044	0.043				
R-squared					0.050	0.048	0.045	0.043

Standard errors in parentheses;

*** p < 0.01,

** p < 0.05,

* p < 0.1; “Yes” means the variable is added to the model.

Similarly, compared with individuals who lived with their parents, individuals who did not live with their parents during the ages of 0–6 had significantly worse mental health in adulthood (coefficient = 2.632, *p* < 0.01). A similar situation occurred during the ages of 7–12 (coefficient = 2.178, *p* < 0.01), 13–15 (coefficient = 1.355, *p* < 0.01), and 16–18 (coefficient = 0.696, *p* < 0.05).

### Sub-group regression by gender

Owing to the gender heterogeneity of our survey sample, we further explored the influence of living without parents at different age stages from the perspective of gender difference. Tables 3, 4 report the effects, separately for adult males and females.

**Table 3:** Influences of living without parents at different stages before adulthood on individual physical health in adulthood between different genders, China, 2018.

**Variables**	**Physical health (ordered probit)**							
	**(1) Male**	**(2) Female**	**(3) Male**	**(4) Female**	**(5) Male**	**(6) Female**	**(7) Male**	**(8) Female**
Living without parents (0–6 years)	−0.161^*^	−0.285^***^						
	(0.093)	(0.088)						
Living without parents (7–12 years)			−0.114	−0.217^***^				
			(0.091)	(0.084)				
Living without parents (13–15 years)					−0.052	−0.120		
					(0.078)	(0.076)		
Living without parents (16–18 years)							0.006	−0.031
							(0.067)	(0.063)
Control variables	Yes	Yes	Yes	Yes	Yes	Yes	Yes	Yes
Region	Yes	Yes	Yes	Yes	Yes	Yes	Yes	Yes
*n*	1,574	1,890	1,574	1,890	1,574	1,890	1,574	1,890
Pseudo R^2^	0.046	0.057	0.046	0.056	0.046	0.055	0.045	0.054

Standard errors in parentheses;

*** p < 0.01,

* p < 0.1; “Yes” means the variable is added to the model.

**Table 4:** Influences of living without parents at different stages before adulthood on individual mental health in adulthood between different genders, China, 2018.

**Variables**	**Mental health (OLS)**							
	**(1) Male**	**(2) Female**	**(3) Male**	**(4) Female**	**(5) Male**	**(6) Female**	**(7) Male**	**(8) Female**
Living without parents (0–6 years)	2.014^***^	3.246^***^						
	(0.689)	(0.651)						
Living without parents (7–12 years)			1.793^***^	2.634^***^				
			(0.672)	(0.621)				
Living without parents (13–15 years)					0.971^*^	1.755^***^		
					(0.578)	(0.566)		
Living without parents (16–18 years)							0.475	0.999^**^
							(0.498)	(0.469)
Control variables	Yes	Yes	Yes	Yes	Yes	Yes	Yes	Yes
Region	Yes	Yes	Yes	Yes	Yes	Yes	Yes	Yes
*n*	1,574	1,890	1,574	1,890	1,574	1,890	1,574	1,890
R-squared	0.045	0.071	0.044	0.067	0.041	0.063	0.040	0.061

Standard errors in parentheses;

*** p < 0.01,

** p < 0.05,

* p < 0.1; “Yes” means the variable is added to the model.

Table 3 shows that the effect on adult physical health of living without parents during the age range 0–6 was statistically significant and negative for both males (coefficient = −0.161, *p* < 0.1) and females (coefficient = −0.285, *p* < 0.01). It suggests that compared with individuals who lived with their parents, both males and females who did not live with their parents during ages 0–6 had significantly worse physical health in adulthood. However, for the age range 7–12, living without parents had a significant and negative influence on physical health of females (coefficient = −0.217, *p* < 0.01) in adulthood, but not on that of males. Furthermore, for the age range 13–18, living without parents had no significant effect on the physical health of males nor females in adulthood. The results indicate that, in terms of long-term physical health outcomes, children need parents more in their early years than when they are older, and that females need the presence of parents for longer than males during their minor years.

In terms of mental health, columns 1, 3, and 5 of Table 4 show that, before the age of 15, living without parents had a significant negative effect on males' mental health in adulthood. Males who did not live with their parents before the age of 15 had greater stress and worse mental health in adulthood than those who lived with their parents before the age of 15. This effect became statistically insignificant in the age 16–18 range, as shown in column (7). For females, it can be seen from columns 2, 4, 6, and 8 of Table 4 that, for all pre-adulthood age ranges (0–18 years), living without parents had a significant negative influence on mental health in adulthood. Compared with females who lived with their parents, females who did not live with their parents at all age stages of pre-adulthood had greater stress and worse mental health in adulthood.

From the influential coefficient, Tables 3, 4 show that, at each age stage, living without parents before adulthood had a greater negative effect on physical and mental health for females in adulthood than for males.

In terms of age distribution, the 0–6 age range was the only one showing a significant negative effect on the physical health of males in adulthood; after the age of 6, the effect was no longer significant. For females, living without their parents had a significant negative effect on their physical health in adulthood until the age of 12; after the age of 12, the effect became statistically insignificant. In terms of mental health, living without parents had a significant negative effect on males in adulthood until the age of 15; after the age of 15, this effect became statistically insignificant. However, during all pre-adulthood age ranges (0–18), living without parents had a significant negative effect on females' mental health in adulthood.

### Dealing with self-selection bias

Table 5 shows the effects of living without parents at the ages of 0–6, 7–12, 13–15, and 16–18 on individual physical and mental health in adulthood by adopting four types of matching methods: nearest-neighbor matching with caliper matching, radius matching, kernel matching, and local-linear regression matching.

**Table 5:** Propensity score matching estimation of the effects of living without parents at the ages of 0–6/7–12/13–15/16–18 on individual physical and mental health in adulthood, China, 2018.

**Method**		**Nearest neighbor**	**Radius**	**Kernel**	**Local linear regression**
0–6 years	Physical health (ATT)	−0.215^***^	−0.201^***^	−0.192^***^	−0.203^***^
		(−3.92)	(−4.04)	(−3.89)	(−3.19)
	Mental health (ATT)	0.264^***^	0.276^***^	0.277^***^	0.278^***^
		(4.25)	(4.79)	(4.84)	(3.97)
7–12 years	Physical health (ATT)	−0.145^***^	−0.151^***^	−0.142^***^	−0.152^***^
		(−2.75)	(−3.15)	(−2.99)	(−2.46)
	Mental health (ATT)	0.259^***^	0.234^***^	0.233^***^	0.234^***^
		(4.56)	(4.41)	(4.42)	(3.59)
13–15 years	Physical health (ATT)	−0.132^***^	−0.112^***^	−0.110^***^	−0.118^**^
		(−2.89)	(−2.70)	(−2.67)	(−2.18)
	Mental health (ATT)	0.174^***^	0.164^***^	0.163^***^	0.170^***^
		(3.56)	(3.59)	(3.61)	(3.06)
16–18 years	Physical health (ATT)	−0.132^***^	−0.112^***^	−0.110^***^	−0.118^**^
		(−2.89)	(−2.70)	(−2.67)	(−2.18)
	Mental health (ATT)	0.174^***^	0.164^***^	0.163^***^	0.170^***^
		(3.56)	(3.59)	(3.61)	(3.06)

ATT means the average treatment effect on treatment. T-statistics are reported in parentheses. The element number of the nearest-neighbor matching with a caliper was 4, the radius was set to 0.01 in radius matching, and kernel matching and local-linear matching used default kernels and bandwidth.

*** p < 0.01,

** p < 0.05.

In the PSM analysis, the values of the average treatment effect on treatment (ATT) in the different matching methods were all significant. The results indicate that, after eliminating observable systematic differences, living without parents at the ages of 0–6, 7–12, 13–15, and 16–18 still had significant and negative effects on individual physical and mental health in adulthood. Thus, the PSM analysis showed that the results of this study are robust.

## Discussion

The Convention on the Rights of the Child of the United Nations Children's Fund pointed out that, the child shall have the right from birth to a name, the right to acquire a nationality and as far as possible, the right to know and be cared for by his or her parents (70). Living with parents and being cared for by parents plays an important role in children's healthy growth and physical and mental health in their adulthood. In this study, we found that compared with children who lived with their parents, individuals who did not live with their parents during their minor years had poorer physical and mental health in adulthood. The results were heterogeneous in age stages and in gender.

Numerous previous studies have shown that the absence of parents has many negative effects on the physical and mental health of minor children (71–73). Children who are not raised by their parents are at higher risk of internet addiction, depression, anxiety, loneliness, suicidal ideation, drug abuse, wasting, stunting, and sickness (71–73). Further, existing studies have found that the effect of parental absence is far-reaching (24–28). Consistently, our empirical results in this study support the above conclusions. The results showed that in terms of physical and mental health, the effect of parental absence on their children is not only immediate, but also into adulthood. Therefore, our general conclusion is that the presence or absence of parents in the household as children grow has both current and long-term impacts on physical and mental health.

This study enriches the research on how adverse experiences in pre-adulthood have negative effects on individuals in adulthood (74). Living with parents is beneficial, and arguably the most important support for children as they grow up (75, 76). From this point of view, not living with parents while growing up can be regarded as an adverse experience of minors. Immediate negative impacts include malnutrition and autism (71–73), but long-term negative effects on physical and mental health in adulthood also are evident as found in this study.

Building on previous studies (39–42, 45, 46), we explored the effects of parental absence based on different age ranges and genders. We found heterogeneous results. Our data showed that as the age of a child increases, the negative effect of living without parents on physical and mental health in adulthood gradually decreases. For younger children, their self-care ability is weaker, they have more emotional needs from adults, and they need more companionship from parents, compared with older children, from the perspective of developmental psychology (77). However, as children growing older, many of them try to become more autonomous from their parents (43). During adolescence and near adulthood, peers, such as close friends or romantic partners, often replace parents and become their attachment figures (43). This enlightens us that the younger the children, the more important it is for their parents to be living with them. This also indicates that parents' intervention in children's health should start from the early stage of their children's life.

We also found that the negative long-term impact of parental absence on physical and mental health is greater for girls than for boys. It implies that in the process of growing up, girls need parents' company more than boys. Therefore, it is particularly important for parents to accompany the growth of their female children. China has a historical tradition of prioritizing boys over girls, and compared to boys, girls may have poor access to parents' care, education, and health services (78). If the parents are absent, the children may be taken care of by their grandparents, who have a more traditional idea of valuing boys over girls, and the girls are less well cared for. Therefore, the absence of parents has a greater impact on girls than boys. Thus, government and non-governmental organizations should formulate relevant policies and increase support for girls' parents to ensure that they are not separated from their minor children. In the long run, strengthening the publicity and education of gender equality, and giving incentive policies, are crucial for girls to get better care from their elders.

The findings of our study have strong practical significance for China. According to the Office of the Leading Group of the State Council for the Seventh National Population Census, as of 2020, a total of 6.436 million children in rural China were left behind when their parents moved from the country to cities to work (29). These children were separated from their parents for most of the year and lived with other relatives, mainly grandparents, with some even living alone (79). Our data analysis confirmed the results of previous studies, which showed that the absence of parents can have a negative effect on the physical and mental health of minors (71–73), and the negative effects can continue into adulthood (24–28). Therefore, it should be the direction of policy efforts to avoid the separation of parents and young children as much as possible.

The decision of parents to separate from their young children is undoubtedly difficult. It is not only an individual decision, but also related to the institutional nature of this issue. The government should make feasible arrangements to reduce the need to separate minor children from their parents. Non-governmental organizations and citizens can also play an active role in creating a social consensus that parents should stay with their minor children. A suboptimal strategy is to provide early intervention for children living without parents, in an attempt to prevent current and future physical and mental health issues.

Our study had some limitations that deserve mention. First, the data regarding who the respondents lived with before adulthood were obtained through the respondents' recall. Because human memory is known to be unreliable, the data may not completely accurately reflect the truth. If future research could track and monitor who minors live with until they reach adulthood, the conclusions could be more objective. Second, the mechanism influencing the long-term impacts of the absence of parents in childhood is still not clear. There is opportunity for future research in this direction. Third, the measurement of physical health in this study relied on one item of self-rated physical health, which may result in measurement bias. For this reason, future studies can use more objective measurements of individuals' physical health. Forth, previous studies have found that father and mother may play heterogeneous roles in children's psychological adjustment; however, restricted by the data availability, we cannot investigate the heterogeneous effects of living without father or mother on children's health in adulthood. Thus, the future study can consider testing the heterogeneous effects of living without father or mother.

## Conclusions

Employing data from a nationwide survey in China, this study analyzed how living without parents at different stages of childhood and adolescence affects an individual's physical and mental health in adulthood. Although the results were heterogeneous at different age stages and for different genders, our analysis showed that growing up without the presence of parents in the household can have a significant negative effect on the physical and mental health of individuals in adulthood. Therefore, the presence of a parent is important for children's health, and has a long-term effect. Future research exploring the mechanism of this effect will be key to furthering our understanding of the long-term effect of lack of parental companionship during childhood.

## Data availability statement

The raw data supporting the conclusions of this article will be made available by the authors, without undue reservation.

## Author contributions

YJ developed the method, wrote the results and discussion, and modified and edited the whole manuscript. HX wrote the literature review, theoretical analysis, results, and modified and edited the whole manuscript. FY proposed the idea of this paper, provided guidance in the theory, modified and edited the whole manuscript, and played the role of supervisor. All authors have read and agreed to the published version of the manuscript.
